# Spontaneous bilateral peripapillary, subhyaloid and vitreous hemorrhage with severe anemia secondary to idiopathic thrombocytopenic purpura

**DOI:** 10.4103/0301-4738.62651

**Published:** 2010

**Authors:** Ajit Babu Majji, Kapil Bhatia, Annie Mathai

**Affiliations:** Smt. Kanuri Shanthamma Center for Retina Vitreous Diseases, L V Prasad Eye Institute, Kallam Anji Reddy Campus, Banjara Hills, Hyderabad-500 034, India

**Keywords:** Anaemia, idiopathic thrombocytopenic purpura, peripapillary hemorrhage, vitreous hemorrhage

## Abstract

A 42-year-old female presented to us with a complaint of sudden painless loss of vision in both eyes of three days duration. Visual acuity was 20/100 for distance in both eyes. Fundus examination showed bilateral peripapillary hemorrhages, with subhyaloid and vitreous hemorrhage in both eyes. Hematological investigations revealed hemoglobin (HB 7 gm %) and severe thrombocytopenia (12,000/ ul). She was referred to a hematologist where a diagnosis of idiopathic thrombocytopenic purpura (ITP) was made. She was treated for systemic condition with regular ophthalmic follow-up. Over the next nine months, retinal hemorrhages completely resolved and the patient regained her visual acuity. The purpose of this case report is to highlight the clinical presentation of severe anemia, which is different from previous reports and the role of an ophthalmologist in first detecting the Idiopathic thrombocytopenic purpura (ITP), which led to successful recovery.

Ocular manifestations of severe anemia include conjunctival pallor and hemorrhages, retinal hemorrhages, tortuous retinal veins, cotton-wool exudates and disc edema, flame-shaped hemorrhages being the commonest type of hemorrhage followed by sub-hyaloid hemorrhage.[1] However, severe bilateral peripapillary hemorrhages have not been reported in literature. Idiopathic thrombocytopenic purpura (ITP) is an autoimmune disease in which antibodies directed against one's own platelets cause their peripheral destruction, resulting in a low platelet count and, occasionally, bleeding complications. Such patients can develop severe grades of anemia, with resultant ocular manifestations. Ophthalmic involvement is exceptionally rare.[2] Severe anemia, when associated with thrombocytopenia can frequently result in ocular manifestations. We present a patient of ITP with severe thrombocytopenia and anemia presenting with sudden painless loss of vision due to bilateral peripapillary, subhyaloid and vitreous hemorrhage.

## Case Report

A 42-year-old lady presented to us on 11.09.2008 with complaint of sudden painless loss of vision in both eyes. She had a history of severe menorrhagia for the past two months. No history of head or ocular trauma was present. She was not a hypertensive or diabetic. Her best corrected visual acuity was 20/100 in both eyes. Anterior segment examination was normal. On fundus examination, both eyes showed peripapillary retinal hemorrhages, subhyaloid and vitreous hemorrhage [Fig. 1 a, b]. An initial impression of anemic retinopathy was made. She was advised hematological evaluation, which revealed hemoglobin 7.2 gm %, RBC count was 2.3 lakh/ cu mm, platelets 12,000 / ul, rest of blood profile was normal (bleeding time, clotting time, prothrombin time and partial thromboplastin time). She was referred to a hematologist, where peripheral smear showed megalokaryocytes and lymphocytes. A bone marrow biopsy confirmed the diagnosis of ITP. Antinuclear antibodies and anti cardiolipin antibodies were negative. She was infused with single donor platelets and IV immunoglobulins till her platelets level returned to normal. She was started on systemic steroids 1 mg/kg/day and hematinics. With this treatment her hemoglobin and platelets attained normal levels. She was under regular ophthalmic evaluation. Her retinal hemorrhages showed complete resolution of peripapillary, retinal and vitreous hemorrhages [Fig. 2a, b]. At final follow-up visit of nine months, her best corrected visual acuity in right eye was 20/30, while in left eye was 20/50.

**Figure 1a:**
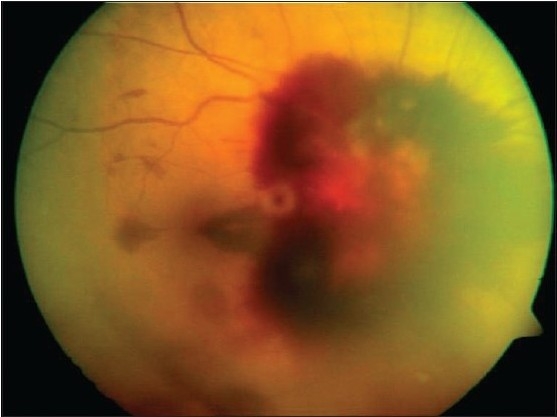
Fundus photographs of right eye showing peripapillary, subhyaloid, vitreous hemorrhage and several flame shaped hemorrhages obscuring the view of the optic disc. Note vessels are of normal caliber and not dilated and tortuous

**Figure 1b:**
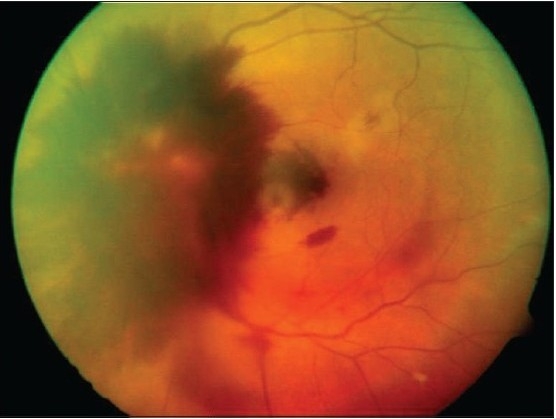
Fundus photographs of left eye showing similar peripapillary, subhyaloid, vitreous hemorrhage and several flame shaped hemorrhages obscuring the view of the optic disc. Vessels are not dilated and tortuous

**Figure 2a:**
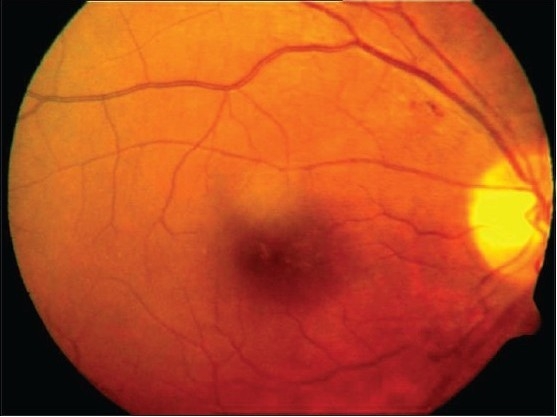
Fundus photograph at 9 months follow-up with complete resolution of hemorrhages

**Figure 2b:**
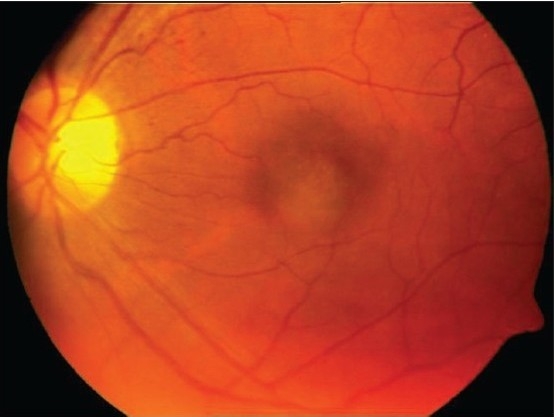
Fundus photograph of left eye at 9 months follow-up with complete resolution of hemorrhages

## Discussion

Ocular manifestations of severe anemia have been increasingly recognized and anemia of varied reasons can result in different ocular manifestations, flame-shaped hemorrhages being the commonest type of hemorrhage followed by sub-hyaloid hemorrhage.[1] Severity of retinal manifestations in anemia depends upon severity of anemia. Our case had severe anemia but associated venous tortuosity and cotton-wool spots were absent. This led to suspicion of associated problems. A systemic work up, revealed diagnosis of ITP, an autoimmune disease, in which antibodies directed against one's own platelets cause their peripheral destruction, resulting in a low platelet count and, occasionally, bleeding complications. Such patients can develop severe grades of anemia, with resultant ocular manifestations and decrease in vision, which can often be bilateral. However ophthalmic involvement is exceptionally rare.[2] Hemorrhagic ophthalmic manifestations associated with ITP include vitreous hemorrhage associated with intracranial bleeding in a Terson type phenomenon,[3] hemorrhage within the optic tract,[4] nonarteritic anterior ischemic optic neuropathy[5] and subconjunctival hemorrhage.[6] Thrombocytopenia alone, even severe (a platelet count <50 000), is rarely sufficient to cause significant retinal hemorrhage. However, thrombocytopenia combined with anemia is a known risk factor, and retinal hemorrhages in association with ITP have only been reported to occur with concurrent severe anemia.[7] There are a few isolated case reports in literature describing similar retinal hemorrhages associated with ITP. Meyer *et al.*[8] described similar retinal findings associated with ITP, but the patient had co-existent diabetic retinopathy. Okuda *et al.*[9] and Paulin *et al.*[10] also described similar findings. In all three reports, patients had to undergo splenectomy along with medical management. In addition, vitrectomy was done for vitreous hemorrhage by Okuda *et al*. In contrast, our patient was managed only by managing systemic factors without any invasive procedure.

So, as clearly demonstrated in our patient, ophthalmic manifestations do not need any specific treatment other than controlling ITP and associated anemia. Good comprehensive examination of the patient with a high index of suspicion can clinch a the systemic diagnosis, and control of systemic parameters will help improve retinopathy associated with ITP and severe anemia.
